# Shear Wave Elastography: A Reliable Secondary Parameter for Diagnosing Biliary Atresia in Infants With Neonatal Cholestasis

**DOI:** 10.7759/cureus.37911

**Published:** 2023-04-20

**Authors:** Avinash D Gautam, Rajanikant R Yadav, Moinak S Sarma, Ankur Mandelia, Vinita Agrawal, Richa Lal, Archna Gupta

**Affiliations:** 1 Department of Radiodiagnosis, Sanjay Gandhi Postgraduate Institute of Medical Sciences, Lucknow, IND; 2 Department of Pediatric Gastroenterology, Sanjay Gandhi Postgraduate Institute of Medical Sciences, Lucknow, IND; 3 Department of Pediatric Surgery, Sanjay Gandhi Postgraduate Institute of Medical Sciences, Lucknow, IND; 4 Department of Pathology, Sanjay Gandhi Postgraduate Institute of Medical Sciences, Lucknow, IND

**Keywords:** neonatal cholestasis, pediatric ultrasonography, triangular cord sign, biliary atresia, ultrasonography (usg), shear-wave elastography

## Abstract

Objective

In this study, we aimed to optimize various grayscale, Doppler, and elastography parameters and evaluate their diagnostic performance in the preoperative diagnosis of biliary atresia (BA).

Materials and methods

A total of 158 infants aged <6 months with neonatal cholestasis (NC) were enrolled in the study and sonography was performed after four hours of fasting. For comparison of elastography, 31 exclusively age-matched controls, not suffering from liver disease, were included separately. Triangular cord and gallbladder (GB) parameters were considered as primary parameters, while right hepatic artery (RHA) caliber, RHA-to-right portal vein (RPV) ratio, hepatic subcapsular flow (HSF), and shear wave elastography (SWE) were considered as secondary parameters. Diagnosis of infants with BA was confirmed on histopathology. Data were presented as mean ±standard deviation (SD) and frequency. Differences between groups were compared using the Chi-square test and the unpaired student t-test. Receiver operating characteristic (ROC) curve analysis was done for individual ultrasound/Doppler/SWE parameters to calculate the optimal cutoff value. Sensitivity, specificity, positive predictive value (PPV), negative predictive value (NPV), and accuracy were calculated for each parameter and their combinations.

Results

Of the primary parameters, GB contractility index (CI) and length showed the highest sensitivity and specificity respectively. A cutoff of 14 kPA was derived for SWE for the diagnosis of BA. Among secondary parameters, SWE had the best diagnostic performance, better than even the individual primary parameters. A combination of primary parameters with SWE in series showed the highest accuracy.

Conclusion

Among secondary parameters, elastography can prove to be highly useful. The highest accuracy in diagnosing BA can be obtained by combining primary parameters with SWE.

## Introduction

Neonatal cholestasis (NC) is defined as impaired bile flow through the biliary system resulting in the accumulation of biliary metabolites in the blood as well as in extrahepatic tissues. The incidence of NC is estimated to be approximately one in 2500 live births [1]. Although there are numerous causes for this condition, biliary atresia (BA) and idiopathic neonatal hepatitis are seen in 60-90% of infants with NC [2]. These two entities have similar clinical and biochemical characteristics. However, they have completely different pathogenesis and management approaches. Therefore, it is very important to accurately diagnose these conditions.

BA is an obliterative cholangiopathy, which, if left untreated, can progress to cirrhosis and end-stage liver failure. The outcome of portoenterostomy is dependent on the age of the patient at surgery and surgical expertise. Currently, the gold standard for the diagnosis of BA is laparotomy with intraoperative cholangiography [3]. However, preoperative diagnosis can also be done using hepatobiliary scintigraphy and percutaneous liver biopsy. Recent studies show a shift towards percutaneous biopsy for confirming preoperative diagnosis [4].

Diagnosing BA at the earliest is critical because Kasai portoenterostomy when performed earlier than 60 days of age establishes adequate bile flow as compared to when performed later. Hence, BA requires early diagnosis for better patient outcomes [3]. In previously published studies, several sonographic, Doppler, and elastography parameters have been used for the preoperative diagnosis of BA with varying success [5].

The purpose of our study was to optimize various grayscale, Doppler, and elastography parameters and evaluate their diagnostic performance in the preoperative diagnosis of BA.

## Materials and methods

Study design and setting

After obtaining approval from the institutional ethics committee, 171 infants aged <6 months with NC were enrolled in the study in the Department of Radiodiagnosis, Sanjay Gandhi Postgraduate Institute of Medical Sciences, Lucknow, India. Details of clinical history, physical examination, laboratory findings, and other investigations were recorded. Sonography was performed on all infants after four hours of fasting, without sedation. Sonography examinations were performed on Aixplorer® (SuperSonic Imagine, Aix-en-Provence, France) using 1-6 MHz single crystal curved XC6-1, 5-18 MHz super linear SL18-5 and 2-10 MHz super linear SL10-2 probes. For comparison of elastography parameters, 31 age-matched controls not suffering from liver diseases were included after taking consent from their guardians.

Parameters assessed during ultrasound examination

The following parameters were analyzed during the ultrasound examination:

Liver Size

The liver size was measured on a longitudinal scan in the midaxillary line.

Triangular Cord Sign

Triangular or tubular echogenic density anterior to the right portal vein (RPV) just distal to the portal vein bifurcation on transverse scanning.

Gallbladder (GB)

GB was evaluated for any irregularity/discontinuity of mucosa or any irregularity in contour, length, prefeed, and 30-minute post-feed GB volume for assessing contractility Index (CI). 

*Right Hepatic Artery (RHA)* and *RPV*

The parameters were measured at the level just proximal to the division of the RPV into anterior and posterior branches from the inner wall to the outer wall. Extension of hepatic artery flow to the hepatic surface under color Doppler was recorded as the presence of the hepatic subcapsular flow (HSF).

Spleen 

Spleen was measured along its long axis from the upper pole to the lower pole. A special note of splenunculi was made.

Cyst at Porta

Cyst at porta, if present, was evaluated for its size, presence of sludge inside, communication with GB, and intrahepatic biliary radicle dilatation.

Shear Wave Elastography (SWE)

SWE of the infants suffering from NC was recorded and compared with 31 other age-matched controls. SWE measurements were obtained during quiet breathing without sedation. The rectangular sample frame was approximately 15 x 15 mm in size, with a circular region of interest of approximately 8-10 mm, avoiding vessels and GB. Hepatic Young's modulus was obtained in the form of an SWE map in kPa. A mean of four values was recorded.

Statistical analysis

Statistical analysis was performed using IBM Statistics version 21 (IBM Corp., Armonk, NY). The normality of the continuous variables was tested using the Shapiro-Wilk test. Continuous variables were presented as mean ±standard deviation (SD) while categorical variables were presented in frequency. Chi-square and Fisher’s exact tests were used to determine the significance of qualitative data between BA and non-BA groups. The unpaired student t-test was used to compare normally distributed continuous variables. A p-value <0.05 was considered statistically significant. The receiver operating characteristic (ROC) curve for the diagnosis of BA was plotted for individual ultrasound/Doppler/SWE parameters, and the optimized cutoff value was calculated using Youden’s Index. The correlation between age and SWE values was evaluated in the BA group by using bivariate Pearson correlation. Sensitivity, specificity, positive predictive value (PPV), negative predictive value (NPV), and accuracy were calculated for each variable and various combinations after optimizing their values.

## Results

**Figure 1:**
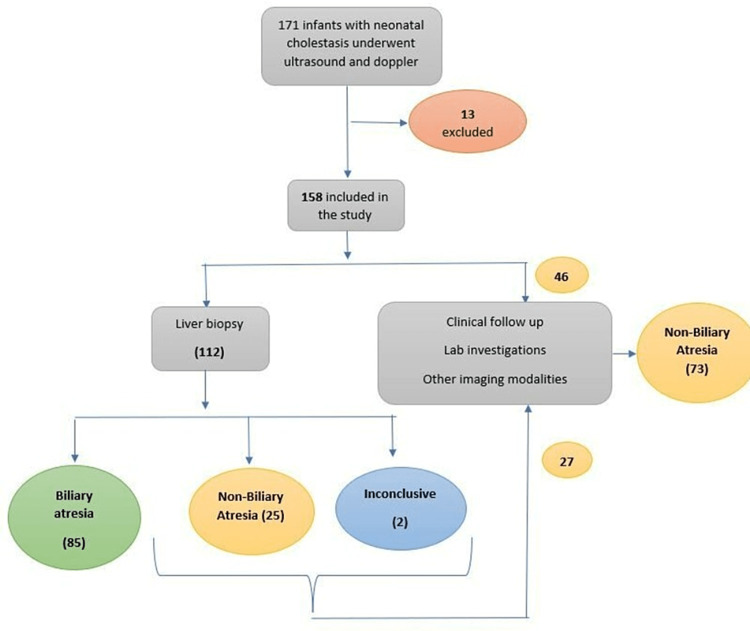
Flow chart demonstrating the process of diagnosis in the study

Out of the 171 examined infants, 13 were excluded, as the diagnosis of 12 infants from the BA group could not be confirmed and one infant was lost to follow-up. Among the remaining 158, 85 were diagnosed with BA based on surgical/histopathological findings. The remaining 73 were diagnosed with other etiologies based on clinical follow-up, imaging, and laboratory studies (Figure 1) (Table 1).

**Table 1:** Final diagnosis of patients in the non-biliary atresia study group

Final diagnosis	Count
Neonatal hepatitis	17
Progressive familial intrahepatic cholestasis	10
Caroli disease	3
Choledochal cyst	3
Niemann-Pick disease	3
Sepsis-induced cholestasis	3
Galactosemia	3
Tyrosinemia	2
Non-syndromic paucity of interlobular bile ducts	2
Alagille syndrome	1
Bile acid synthetic defect	1
Choledocholithiasis	1
Neonatal hemochromatosis	1
Neonatal sclerosing cholangitis	1
Spontaneous bile leak	1
Transient neonatal cholestasis (multifactorial)	21
Total	73

Of the 85 histologically confirmed BA patients, 16 were operated on and two underwent additional magnetic resonance cholangiopancreatography (MRCP).

No significant statistical difference was noted between the demographic characteristics of the BA, non-BA, and control groups (Table 2).

**Table 2:** Demographic details of the patient *Age presented in form of mean ±SD, tested by ANOVA. **Gender presented in the form of ratios, tested by the Chi-square test ANOVA: analysis of variance; SD: standard deviation

Demographic data	Biliary atresia (n=85)	Non-biliary atresia (n=73)	Controls (n=31)	P-value
Age* in months, mean ±SD	2.66 ±1.35	2.52 ±1.804	3.10 ±2.006	0.266
Sex** (F:M)	31:54	21:54	12:19	0.489

Primary parameters

Triangular Cord Sign

The mean triangular cord thickness in the BA group was significantly higher than in the non-BA group. Using ROC curve analysis, the optimal cutoff for the triangular cord was 2.2 mm, which was rounded off to 2 mm, as ultrasound is not sensitive for measuring 0.2 mm.

GB Parameters

GB was not visualized in six cases in the BA group. For evaluation of other parameters, cases with absent GB were excluded and results were obtained (n=152). The mean GB length, prefeed volume, and CI were significantly different between the BA and non-BA groups. Cutoff for GB length and CI were 1.89 cm and 59.5% respectively, which were rounded off to 1.9 cm and 60% respectively. Using optimized cutoffs, GB was defined as abnormal if it was absent or the length was <1.9 cm or the CI was <60%. The diagnostic performance of the abnormal GB was further examined.

Table 3 presents a comparison of the various ultrasound parameters between the BA and non-BA groups. Table 4 shows the optimal cut-off values and area under the curve derived from ROC curves for various parameters.

**Table 3:** Comparison of various ultrasound parameters between the BA and non-BA groups *P<0.05 indicates statistical significance Data presented as mean ±SD; independent samples t-test used BA: biliary atresia; GB: gallbladder; RHA: right hepatic artery; RPV: right portal vein

Parameters	BA group, mean ±SD	Non-BA group, mean ±SD	P-value*
Liver size, cm (n=158)	7.49 ±0.95	6.97 ±0.947	<0.01
Triangular cord, mm (n=158)	3.4 ±2.02	0.65 ±1.38	<0.01
GB length, cm (n=152)	1.66 ±0.735	2.54 ±0.802	<0.01
Contractility index (CI) (n=152)	17.38 ±40.3	62.45 ±31.29	<0.01
Prefeed GB volume, ml (n=152)	0.171 ±0.2	1.005 ±2.5	<0.01
Post-feed GB volume, ml (n=152)	0.131 ±0.18	0.193 ±0.25	0.087
RHA diameter, mm (n=158)	1.86 ±0.446	1.22 ±0.72	<0.01
RPV diameter, mm (n=158)	3.64 ±0.726	3.63 ±0.79	0.934
RHA/RPV diameter ratio (n=158)	0.525 ±0.145	0.346 ±0.12	<0.01
Spleen size, cm (n=158)	6.13 ±1.04	5.96 ±1.05	0.317

**Table 4:** Optimal cutoff and area under the curve derived from ROC curves for various ultrasound parameters *Number in parentheses are 95% confidence intervals GB: gallbladder; RHA: right hepatic artery; ROC: receiver operating characteristic; RPV: right portal vein

Parameter	Area under the curve*	Optimal cutoff	Youden’s Index
Triangular cord, mm (n=158)	0.856 (0.792,0.920)	2.2	0.696
GB length, cm (n=152)	0.797 (0.726,0.868)	1.89	0.483
Contractility index, % (n=152)	0.818 (0.751,0.886)	59.5	0.572
RHA diameter, mm (n=158)	0.859 (0.801,0.918)	1.45	0.616
RHA/RPV diameter ratio (n=158)	0.840 (0.778,0.902)	0.356	0.565

Secondary parameters

RHA

The mean RHA caliber in the BA group was significantly higher than in the non-BA group (Table 3). ROC curve analysis for RHA caliber showed the optimal cutoff to be 1.45 mm (Table 4), which was rounded off to 1.5 mm for statistical analysis.

RHA/RPV Ratio

The mean RHA/RPV ratio in the BA group was significantly higher than in the non-BA group (Table 3). The optimal cutoff for RHA/RPV ratio was 0.356 (Table 4), which was rounded off to 0.4.

HSF 

HSF* *was seen in 47 patients in the BA group and nine patients in the non-BA group.

Elastography

Out of 158 patients with NC, 77 patients (BA: 42, non-BA: 35) underwent SWE examination along with 31 age-matched controls for comparison. The mean of the hepatic Young’s modulus in the BA group was significantly higher than in the non-BA group and the control group (Table 5). Using ROC curve analysis, the cutoff to diagnose BA was 14 kPa, with a sensitivity of 100%, specificity of 90.9%, NPV of 100%, PPV of 87.5%, and accuracy of 94.4%. The diagnostic performance of the SWE cutoff derived in our study was optimal when compared to the individual performance of secondary and even primary parameters. SWE measurements in the BA group when correlated with age showed a moderate positive correlation (r-value=0.491).

**Table 5:** Hepatic Young’s modulus (kPa) descriptive of groups Table showing Mean ±SD of hepatic Young’s modulus in various study groups Means were compared with the ANOVA F test and showed a statistically significant difference (p<0.01) BA: biliary atresia

Groups (n=108)	Mean ±SD (kPa)
BA group (n=42)	19.403 ±2.843
Non-BA group (n=35)	11.155 ±2.956
Controls (n=31)	6.238 ±1.435

Other parameters

A significant difference was noted in mean liver size between the groups. However, no significant difference was noted in mean spleen size and RPV (Table 3). Three cases of polysplenia were seen in the BA group.

In 12 BA cases, an isolated cyst was noted at porta without intrahepatic bile radicle dilatation, sludge, or calculi. The size of the cysts ranged from 0.4 cm to 5.6 cm with a mean size of 2.295 ±2 cm.

Diagnostic Performance of Individual Ultrasound/Doppler/SWE Parameter 

Among primary parameters, GB CI showed the highest sensitivity, while GB length was highly specific. Among primary parameters, the triangular cord was highly accurate in confirming or refuting BA with certainty (Table 6). Among secondary parameters, SWE was highly sensitive and specific, followed by HSF in specificity (Table 6).

**Table 6:** Diagnostic performance of various ultrasound and Doppler parameters with optimized cutoffs *Values for these GB parameters were tested for 152 cases in which GB was visualized. **Values for SWE were tested for 77 cases P-value <0.05 suggests a significant association of the parameter with the BA group NPV: negative predictive value; PPV: positive predictive value; GB: gallbladder; CI: contractility index; RHA: right hepatic artery; RPV: right portal vein; SWE: shear wave elastography

Parameters tested	Sensitivity (%)	Specificity (%)	NPV (%)	PPV (%)	Accuracy (%)	P-value
Triangular cord thickness ≥2 mm (n=158)	81.17	83.5	79.2	85.18	82.27	<0.001
GB length <1.9 cm (n=152)*	62.02	86.3	67.7	83.05	73.68	<0.001
GB morphology (n=152)*	82.27	82.19	81.08	83.3	82.23	<0.001
CI <60% (n=152)*	87.34	69.8	83.6	75.82	78.9	<0.001
Abnormal GB (CI <0.6 or abnormal GB morphology or GB length <1.9 cm or absent GB) (n=158)	91.7	64.38	75	87.03	79.1	<0.001
RHA caliber ≥1.5 mm (n=158)	83.5	78.08	80.2	81.6	81.01	<0.001
RHA/RPV ≥0.4 (n=158)	81.17	71.2	76.4	76.6	76.5	<0.001
Hepatic subcapsular flow (n=158)	55.2	87.6	62.7	71.1	70.2	<0.001
SWE (n=77)**	100%	90.9%	100%	87.5%	94.4%	<0.001

Diagnostic Performance of USG/Doppler/SWE Parameters in Combination

We combined various parameters in parallel and series tests. For the parallel test, if any of the parameters fulfilled the criteria, the test was considered positive. While in series tests, the examination was considered positive only if the criteria for all the parameters were met (Table 7). The combination of triangular cord, abnormal GB, and SWE in series showed the best diagnostic performance (sensitivity: 78.57, specificity: 97.14, NPV: 97.06, PPV: 79.07, and accuracy: 87.01) (Table 7).

**Table 7:** Diagnostic performance of the combination of various parameters in parallel or series arrangements for the diagnosis of biliary atresia P-value <0.05 suggests that the association of groups and outcomes is statistically significant NPV: negative predictive value; PPV: positive predictive value; GB: gallbladder; EARPV: echogenicity anterior to the right portal vein; CI: contractility index; RHA: right hepatic artery; RPV: right portal vein; HSF: hepatic subcapsular flow; SWE: shear wave elastography

Combination of parameters	Arrangement	Sensitivity (%)	Specificity (%)	NPV (%)	PPV (%)	Accuracy (%)	P-value
Abnormal GB or EARPV ≥2 mm (n=158)	Parallel	94.11	56.1	89.1	71.4	71.4	<0.01
Abnormal GB and EARPV ≥2 mm (n=158)	Series	78.8	91.78	78.8	91.7	84.8	<0.01
Abnormal GB or EARPV ≥2 mm or RHA ≥1.5 mm (n=158)	Parallel	98.8	5.4	80	54.9	55.69	0.123
Abnormal GB and EARPV ≥2 mm and RHA ≥1.5 mm (n=158)	Series	74.1	94.52	75.8	94.02	83.5	<0.01
Abnormal GB or EARPV ≥2 mm or RHA ≥1.5 mm or RHA/RPV ≥0.4 (n=158)	Parallel	98.8	38.3	96.5	65.1	70.8	<0.01
Abnormal GB, EARPV ≥2 mm, RHA ≥1.5 mm, and RHA/RPV ≥0.4 (n=158)	Series	67.05	95.8	71.4	95	80.3	<0.01
Abnormal GB or EARPV ≥2 mm or RHA ≥1.5 mm or RHA/RPV ≥0.4 or HSF (n=158)	Parallel	75	38.3	50	65.1	70.8	<0.01
Abnormal GB, EARPV ≥2 mm, RHA ≥1.5 mm, RHA/RPV ≥0.4 and HSF (n=158)	Series	47.05	98.6	61.5	97.5	70.8	<0.01
Abnormal GB or EARPV ≥2 mm or RHA ≥1.5 mm or RHA/RPV ≥0.4 or HSF or SWE (n=77)	Parallel	100	34.3	100	64.62	70.13	<0.001
Abnormal GB, EARPV ≥2 mm, RHA ≥1.5 mm, RHA/RPV ≥0.4, HSF and SWE (n=77)	Series	45.24	100	60.34	100	70.13	<0.001
Abnormal GB or EARPV ≥2 mm or SWE (n=77)	Parallel	100	54.29	72.41	100	79.22	<0.001
Abnormal GB, EARPV ≥2 mm and SWE (n=77)	Series	78.57	97.14	97.06	79.07	87.01	<0.001

## Discussion

BA must be excluded in any infant with NC as the prognosis is improved by early diagnosis and prompt surgery. Ultrasound as a screening modality offers several advantages: it is a widely available, affordable, and radiation-free procedure that can be performed at the bedside.

We classified various ultrasound/Doppler/SWE parameters in this study into two categories: primary parameters (which directly define or quantify abnormalities in the extrahepatic biliary system) and secondary parameters (which are abnormalities in other structures that arise as a consequence of BA). Thus, we considered triangular cord sign, abnormal GB morphology, and decreased contractility of the GB after feeding as primary parameters. Hepatomegaly, enlarged RHA, increased RHA/RPV ratio, and splenomegaly were considered secondary parameters, similar to a previously conducted study by Mittal et al. (2011) [6]. Since elastography is an indicator of liver fibrosis [7], we classified it as a secondary parameter, as hepatic fibrosis is not exclusive to BA.

On comparing primary parameters, the optimized triangular cord sign had the highest accuracy (82.27%) followed by GB morphology (82.23%) for the diagnosis of BA (Table 6). In our study, the absence or non-visualization of GB was always associated with BA, as previously observed by Mittal et al. (2011) [6]. The most sensitive primary parameter was GB CI (87.34%) while the most specific was GB length (86.3%).

**Figure 2:**
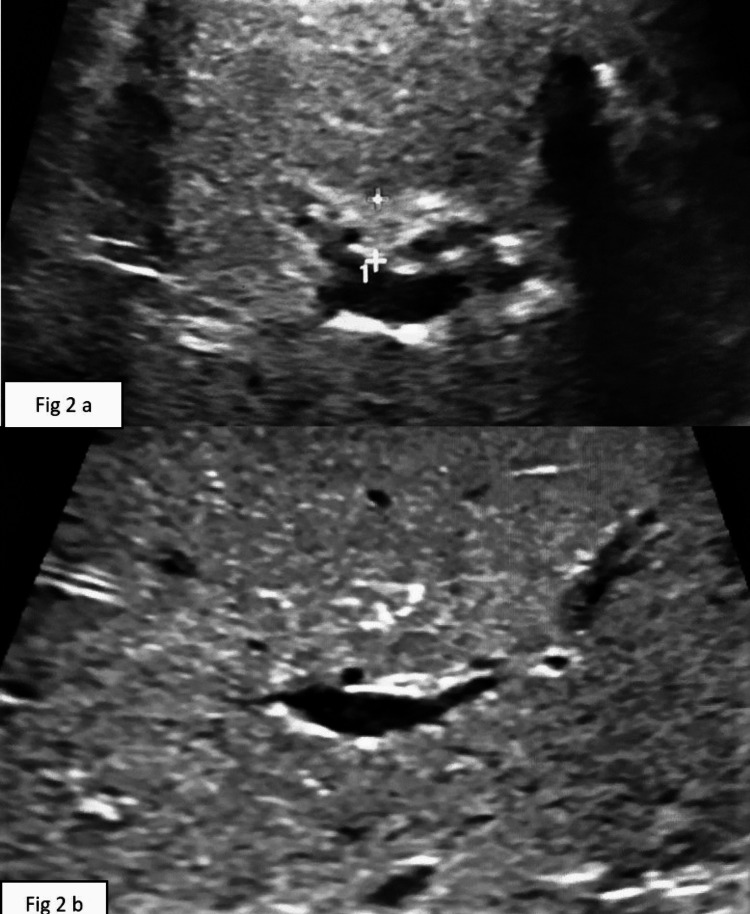
Triangular cord sign a) The image shows echogenicity anterior to the right branch of the portal vein with calipers measuring the thickness (4.2 mm) in a biopsy-proven case of biliary atresia; b) The image shows no measurable echogenicity anterior to the right branch of the portal vein in a 2-month-old infant suffering from idiopathic neonatal hepatitis

In our study, for the triangular cord sign, we measured echogenicity anterior to the RPV just distal to the portal vein bifurcation on transverse scanning (Figure 2), and serial recording was done, which was then analyzed using the ROC curve and a cutoff of 2 mm was obtained, which proved to be highly accurate.

**Figure 3:**
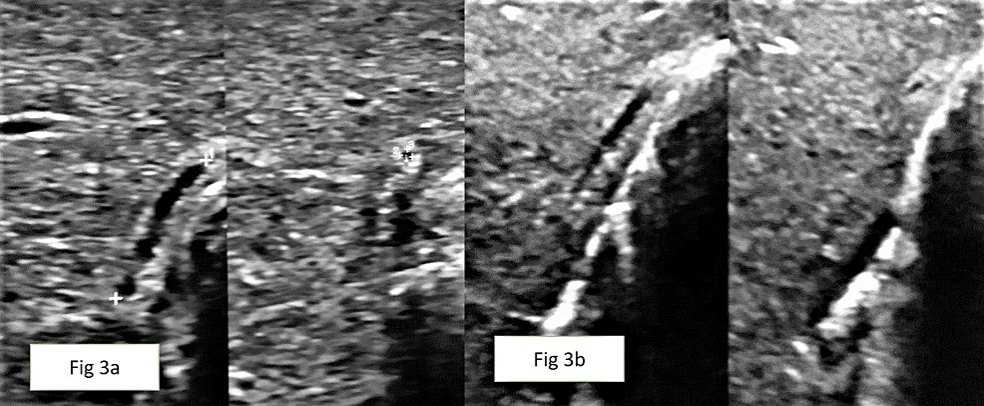
Prefeed (a) and post-feed (b) images in a 2-month-old child suffering from biliary atresia No significant appreciable change in gall bladder volume is seen suggestive of abnormal gall bladder contraction. (Length: 1.2 cm, prefeed volume: 0.15 ml, post-feed volume: 0.12 ml, and contractility index: 20%)

**Figure 4:**
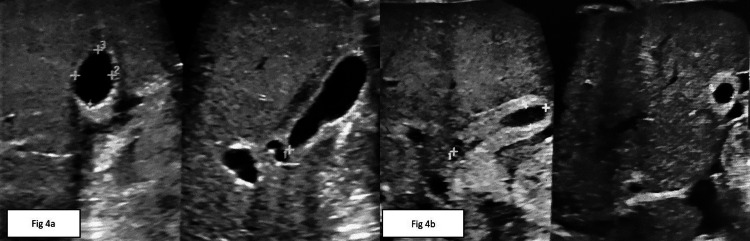
Prefeed (a) and post-feed (b) images of a 1-month-old child suffering from idiopathic neonatal cholestasis Normal gall bladder length, morphology, and contraction are seen, suggestive of a normal gall bladder. (Length: 2.2 cm, prefeed volume: 0.99 ml, post-feed volume: 0.6 ml, and contractility index: 69.6%)

GB abnormalities were analyzed under three parameters: GB length, GB morphology, and GB CI (Figures 3, 4). Among these, GB morphology had the highest accuracy (82.23%) for the diagnosis of BA. An optimized cutoff of 1.9 cm for GB length showed a diagnostic performance comparable to previously published studies [5,6,8]. Gallbladder contraction after feeding had been evaluated in a few studies in the literature but no objective cutoff has been defined for CI [5,6,9]. In our study, we measured the prefeed and post-feed volume of GB and calculated the CI. A CI cutoff of 60% proved to be the most sensitive among the other GB parameters in our study, while GB morphology proved to be highly accurate (Table 6). Using the optimized values, we defined GB as abnormal if “it was not visualized, or GB length was <1.9 cm or GB CI was <60%, or GB morphology was abnormal”. The definition of abnormal GB used in our study proved to be highly sensitive (91.7%) for the diagnosis of BA (Table 6).

**Figure 5:**
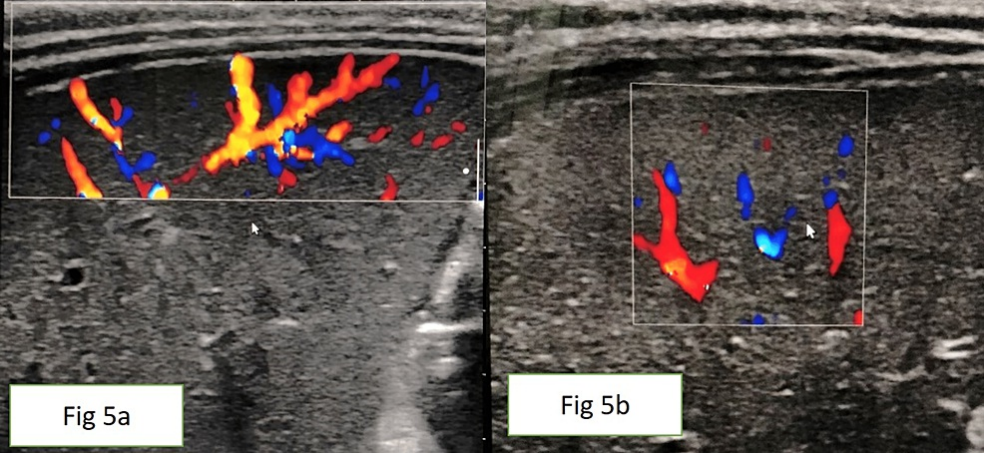
a) Hepatic subcapsular flow (HSF) in biopsy-proven case of biliary atresia; b) Absence of HSF in a case of transient neonatal hepatitis

**Figure 6:**
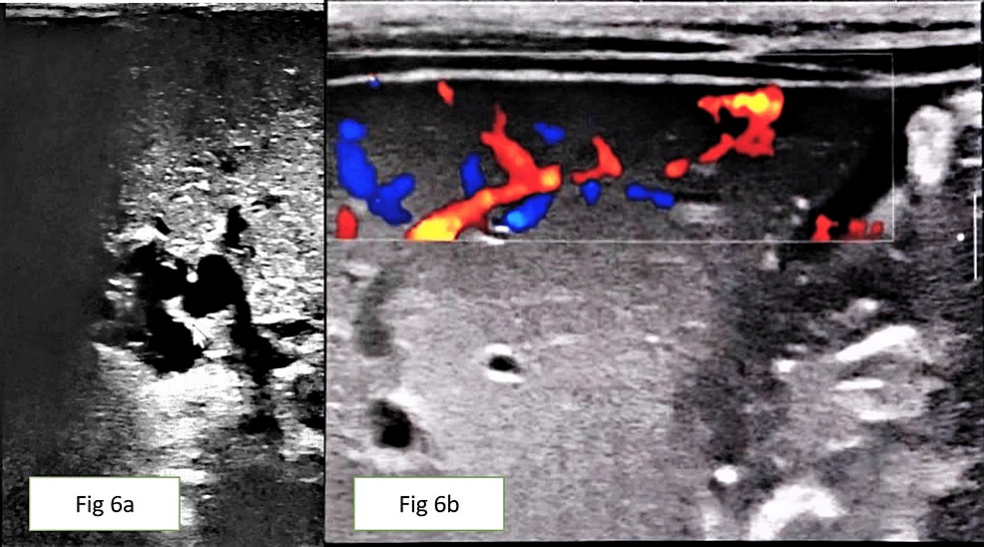
A known case of Caroli's disease: a) Ultrasound image demonstrating multiple intrahepatic cystic dilatations of the biliary tree; b) Doppler image demonstrating the presence of hepatic subcapsular flow

Diagnostic performance for optimized RHA and RHA/RPV (Table 6) was comparable to previously conducted studies [6,10]. However, enlarged RHA was also noted in 16 cases in the non-BA group with etiologies like progressive familial intrahepatic cholestasis (PFIC) (n=4), NH (n=3), and choledochal cyst (n=2). HSF was also recorded in nine infants in the non-BA group with etiologies like PFIC (n=3) and NH (n=2) (Figures 5, 6). Therefore, hepatic arterial parameters should be used adjunctively rather than solely, for the diagnosis of BA. However, as a secondary finding, HSF is quite specific (87.6%) among the rest of the secondary Doppler parameters for the diagnosis of BA (Table 6). RHA enlargement and HSF in BA are postulated to be due to hepatic arteriopathy secondary to hepatic fibrosis [11], compensatory change to improve the biliary tree blood supply secondary to liver cirrhosis, or a vascular malformation [6]. While diagnosing BA, one should always keep in mind that other diseases affecting liver parenchyma may also alter hepatic arterial flow and result in arterial dilatation and increased flow due to hepatic artery buffer response, owing to a decrease in portal venous flow due to any cause [12]. However, this theory needs to be further evaluated for infants.

In view of some overlapping of ultrasound/Doppler parameters between BA and non-BA groups, one should always be careful before labeling the diagnosis as BA. Invasive procedures like biopsy or laparotomy may be needed for confirmation. To aid in such situations, elastography can prove to be a useful adjunctive tool [7,13,14].

**Figure 7:**
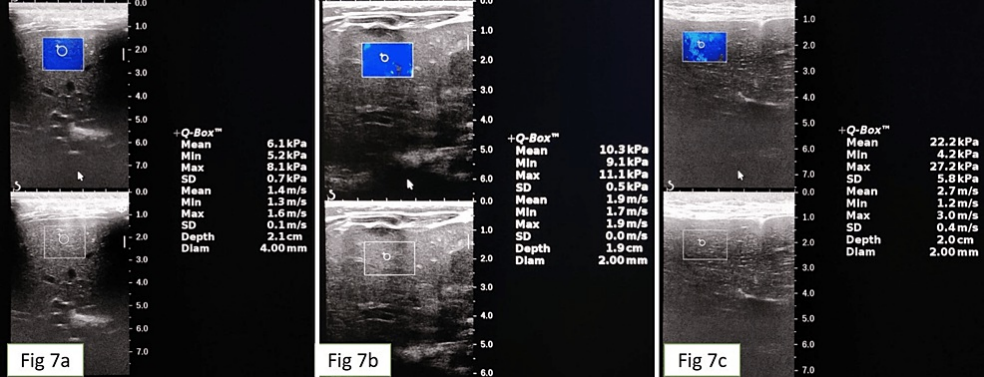
Shear wave elastography (SWE) measurements in a) control, b) neonatal hepatitis, and c) biliary atresia

In our study, 77 out of the total 158 infants with NC underwent elastography, and SWE values were compared with 31 age-matched controls (Table 7). The mean of hepatic Young’s modulus in the BA group was significantly higher than in the non-BA and control groups (Figure 7). A derived cutoff value of 14 kPa showed high sensitivity (100%), specificity (90.9%), and accuracy (94.4%) for the diagnosis of BA, which was comparable to the previously published studies [7,14]. In our study, the diagnostic performance of SWE was better than that of individual secondary and even primary parameters.

Considering the nature of the disease, a test with high sensitivity is needed as a first-line screening tool. In our study, when SWE was not considered, the combination of triangular cord sign, abnormal GB, RHA caliber, and RHA/RPV ratio in parallel had the highest sensitivity (98.8%) for the diagnosis of BA (Table 7). The highest specificity was obtained when a combination of triangular cord, abnormal GB, RHA caliber, RHA/RPV ratio, and HSF was employed in series (98.6%) (Table 7). But none of these combinations had good accuracy.

The combination of primary parameters (triangular cord and abnormal GB) with SWE in series showed the best diagnostic performance with the highest accuracy (87.01%), which was higher than all the combinations of ultrasound/Doppler/SWE parameters in our study (Table 7).

The differences in mean SWE values obtained between the three groups (Table 6) point towards the usefulness of elastography as an adjunctive tool for the early diagnosis of BA when in doubt. A moderate positive correlation of liver stiffness with age observed in our study can be attributed to the progression of biliary cirrhosis in infants with BA.

## Conclusions

BA has a grave prognosis if the diagnosis is delayed. A combination of ultrasound/Doppler/SWE can substantially expedite the workup of suspected BA cases. The highest accuracy in diagnosis can be obtained by combining primary abnormalities in BA (i.e., modified triangular cord and abnormal GB) with SWE in series. Secondary abnormalities in BA such as RHA caliber, RHA/RPV, HSF, and SWE overlap between BA and non-BA groups and hence should always be used adjunctively in the diagnosis of BA. SWE with a cutoff of 14 kPA has the best individual diagnostic performance among secondary parameters and can prove to be highly accurate (87.01%) and specific (97.14%) for the diagnosis of BA when used in series with primary parameters alone.
